# Integrated Analysis of Single-Cell and Bulk RNA-Sequencing Reveals a Tissue-Resident Macrophage-Related Signature for Predicting Immunotherapy Response in Breast Cancer Patients

**DOI:** 10.3390/cancers14225506

**Published:** 2022-11-09

**Authors:** Zi-An Xia, You Zhou, Jun Li, Jiang He

**Affiliations:** 1 Department of Integrated Traditional Chinese and Western Medicine, Xiangya Hospital, Central South University, Changsha 410008, China; 2 National Clinical Research Center for Geriatric Disorders, Xiangya Hospital, Central South University, Changsha 410008, China; 3 Department of Pathology, Tongji Medical College Union Hospital, Huazhong University of Science and Technology, Wuhan 430022, China; 4 Department of Nuclear Medicine, Peking University Shenzhen Hospital, Guangdong 518036, China; 5 Department of Oncology, Xiangya Cancer Center, Xiangya Hospital, Central South University, Changsha 410008, China; 6 Key Laboratory of Molecular Radiation Oncology Hunan Province, Changsha 410008, China

**Keywords:** breast cancer, immune checkpoint therapy, tissue-resident macrophages, single-cell RNA-sequencing

## Abstract

**Simple Summary:**

Immune checkpoint therapy (ICT) has proven to be a promising therapeutic approach to breast cancer (BC), but most patients with BC do not respond to ICT and there are no validated predictive biomarkers. Therefore, it is urgently necessary to identify a valuable biomarker for predicting ICT outcomes of BC patients. In this study, we performed scRNA-seq analysis and identified five tissue-resident macrophages (RTM) clusters with a mixed phenotype of M1-M2 macrophages. An integrated analysis of multi-omics data showed RTM clusters were characteristic of an elevated inflammatory response and reactive oxygen species pathway, and positively correlated with T cell cytotoxicity and infiltration of CD8+ T cells and CD8+ T cells, which is indicative of sensitivity to ICT. Therefore, the RTM clusters may serve as a valuable tool for clinical decision making in BC patients receiving ICT.

**Abstract:**

Immune checkpoint therapy (ICT) is among the widely used treatments for breast cancer (BC), but most patients do not respond to ICT and the availability of the predictive biomarkers is limited. Emerging evidence indicates that tissue-resident macrophages (RTMs) inhibit BC progression, suggesting that their presence may predict immunotherapy response. A single-cell RNA-sequencing analysis of BC samples was performed to identify five RTM clusters with a mixed phenotype of M1-M2 macrophages. The comprehensive results showed that a high score of each RTM cluster was associated with a high infiltration of CD8+ T cells, M1 macrophages, and dendritic cells, and improved overall survival. In addition, a low score of each RTM cluster was associated with a high infiltration of M0 macrophages, naïve B cells and Tregs, and poor overall survival. Gene signatures from each RTM cluster were significantly enriched in responders compared with nonresponders. Each RTM cluster expression was significantly higher in responders than in nonresponders. The analyses of bulk RNA-seq datasets of BC samples led to identification and validation of a gene expression signature, named RTM.Sig, which contained the related genes of RTM clusters for predicting response to immunotherapy. This study highlights RTM.Sig could provide a valuable tool for clinical decisions in administering ICT.

## 1. Introduction

Tissue-resident macrophages (RTMs) are a heterogeneous immune cell population derived from embryonic precursor cells and a part of tumor-infiltrating macrophages [1,2]. MHCII^hi^ CD11b^hi^ macrophages have been defined as RTMs in breast cancer (BC) [3]. Emerging evidence indicates that RTMs exert anti-tumor activity in BC [4], suggesting that their presence might predict immunotherapy response.

Anti-tumor immune checkpoint therapy (ICT) that relieves immunosuppression is a powerful clinical approach [5,6]. Nonetheless, most patients do not respond and the availability of the predictive biomarkers is limited [5,7,8]. Therefore, it is necessary to explore effective predictive biomarkers for ICT response. Previous studies indicate that scRNA-seq significantly promotes the identification of factors underlying the ICT outcomes [9]. Recent studies showed that RTMs increased CD8+ T cell infiltration [4]. This suggests that RTMs could improve the response to ICT and provide potential evidence for predicting the immunotherapy response.

To determine whether RTMs were associated with ICT outcomes, we analyzed the scRNA-seq dataset of BC samples and identified five RTM clusters that played an important role in determining ICT responsiveness. These RTM clusters were characterized by the high expression of genes coding inflammatory response, reactive oxygen species pathway, and interferon response, etc. We then characterized the immune profile of RTM clusters and examined their prognostic ability. Gene signatures from each RTM cluster, excluding RTM_4, were significantly enriched in responders compared with nonresponders. Gene signatures of each RTM cluster were enriched in BC samples from responding patients. The analyses of bulk RNA-seq datasets of BC samples identified and validated a 25-gene expression signature -RTM.Sig- enriched with the related genes of RTM clusters to predict response to ICT. The results showed that the RTM.Sig can precisely predict the ICT outcomes of BC patients compared with the previously reported ICT response signatures, including well-established IMPRES. These findings improve our understanding of RTMs and might improve the clinical diagnosis and treatment strategies of BC.

## 2. Materials and Methods

### 2.1. Study Design

Single-cell RNA-sequencing data (accession number GEO: GSE161529) of BC samples (Table S1) from the initial publication [10] were analyzed to identify RTM clusters. The METABRIC (Molecular Taxonomy of Breast Cancer International Consortium) database (n = 1904) is used to analyze immune characteristics of RTM clusters and the relationship between RTM clusters and prognosis. The analysis of a bulk RNA-seq dataset (accession number: GSE177043 [11]) of pretreatment BC samples with anti-PD1 therapy outcomes (16 responders and 27 nonresponders) developed an ICT response signature—RTM.Sig. To validate the predictive performance of RTM.Sig, three public gene expression datasets of ICT (respectively, accession number: EGAD00001006608, GSE111414, and GSE168204) were analyzed. These three datasets comprised clinical information on anti-PD1 therapy outcomes. The first dataset (EGAD00001006608) [12] comprised pretreatment BC samples from 29 patients (20 responders and 9 nonresponders). The second dataset (GSE111414) [13] consisted of pretreatment lung cancer samples from 20 patients (10 responders and 10 nonresponders). For the third dataset (GSE168204) [14], the bulk RNA-seq data of pretreatment melanoma samples from 27 patients (18 responders and 9 nonresponders) was analyzed.

### 2.2. Immunofluorescence Staining

Immunofluorescence staining was performed according to a previously described protocol [15]. The following antibodies were purchased and used to detect specific proteins: anti-CD68 (mouse, 1:100, ZSGB-BIO, catalog no. ZM-0464) anti-MARCO (rabbit, 1:100, Abcam, catalog no. ab239369), and anti-FOLR2 (rabbit, 1:100, Abcam, catalog no. ab103998).

### 2.3. Quality Control and Cell Type Recognition

A Seurat (version 4.0.4) package in R software (version 4.1.1 https://cran.r-project.org/web/packages/SeuratObject/index.html) was used to analyze the scRNA-seq data [16]. Based on the previous criterion of quality control [10], single cells with < 200 genes or UMI count < 1000 or the percent of mitochondrial genes over 20% of total expressed genes were screened as low-quality cells and eliminated. Eventually, 106,289 filtered cells were obtained for further bioinformatics analysis.

After normalizing the filtered gene-barcode matrix using the “LogNormalize” method, the top 2000 variable genes were selected through the FindVariableFeatures function with the “vst” method in Seurat. Variables of the UMI count and the percentage of mitochondrion-derived UMI counts were removed in the scaling step. Principal component analysis (PCA) was adopted for dimensionality reduction. JackStraw function was used for calculating significant principal components. For establishing the best model, the top number of principal components (PC) was as follows: (i) the cumulative contribution of PCs was greater than 90%; (ii) the contribution of PC itself to the total variance was less than 5%; and (iii) the difference between two consecutive PCs was less than 0.1%. Subsequently, 2D t-SNE and UMAP were used to observe the primary cell clusters. DEGs in each cluster were acquired using the FindMarkers function in the Seurat package. Thereafter, the major cell types were recognized based on the previously reported markers (Table S1). Among these, three immune cell types, including macrophages, B cells, and T cells, respectively, were extracted for downstream clustering analysis. These data were processed and analyzed as described above.

### 2.4. Functional Enrichment Analysis

A clusterProfiler R package (version 4.0.5) was used for KEGG enrichment analysis based on the DEGs [17]. Subsequently, its gseKEGG function was performed for GSEA analysis. Meanwhile, a gene set variation analysis (GSVA) package (version 1.40.1) was used for calculating GSVA scores of 50 hallmark gene sets from the Molecular Signatures Database (MSigDB) [18]. All threshold values in these packages were set to default as described in its vignette. 

### 2.5. Comprehensive Analysis of Molecular and Immune Characteristics in Different Subtypes of Each RTM Cluster

To identify the immune characteristics of each RTM cluster in1904 breast cancer samples, their gene signatures were extracted from the METABRIC dataset to calculate GSVA scores and their expression data were imported into CIBERSORT (https://cibersort.stanford.edu/, (accessed on 25 August 2022), and iterated 1000 times to estimate the relative proportion of 22 types of immune cells. Then, we compared the relative proportions of 22 types of immune cells between the two subtypes of each RTM cluster and the results are presented in a landscape map.

### 2.6. Prognostic Analysis of Each RTM Cluster

To estimate the prognostic value of each RTM cluster, their gene signatures were extracted from the METABRIC dataset to calculate GSVA scores. These data were further loaded into a survival R package (version 3.2.13) for OS analysis. The results were observed using the Kaplan–Meier plots. 

### 2.7. Prediction of ICT Outcomes

Four public gene expression datasets (GSE177043, EGAD00001006608, GSE111414 and GSE168204) with anti-PD1 immunotherapy were downloaded to predict ICT outcomes using a cancer class R package (version 1.36.0) as previously described [19]. The AUC value of receiver-operating-characteristic curves (ROC) was calculated to estimate their predictive capacity.

### 2.8. Immune Checkpoint Analysis

To show the diversity of immune checkpoints in different clusters, their gene expression was extracted from each cluster. Subsequently, the average values of each gene were calculated and loaded into the pheatmap R package (version 1.0.12). The scale was set as a “row”. Eventually, expressions of immune checkpoints were visualized as a heatmap.

### 2.9. Statistical Analysis

Statistical analyses were performed using the R software (version 4.1.1). Table S6 shows the corresponding R codes. All *p* values with less than 0.05 (*p* < 0.05) were considered statistically significant.

## 3. Results

### 3.1. Identification of RTMs in BC

**Figure 1:**
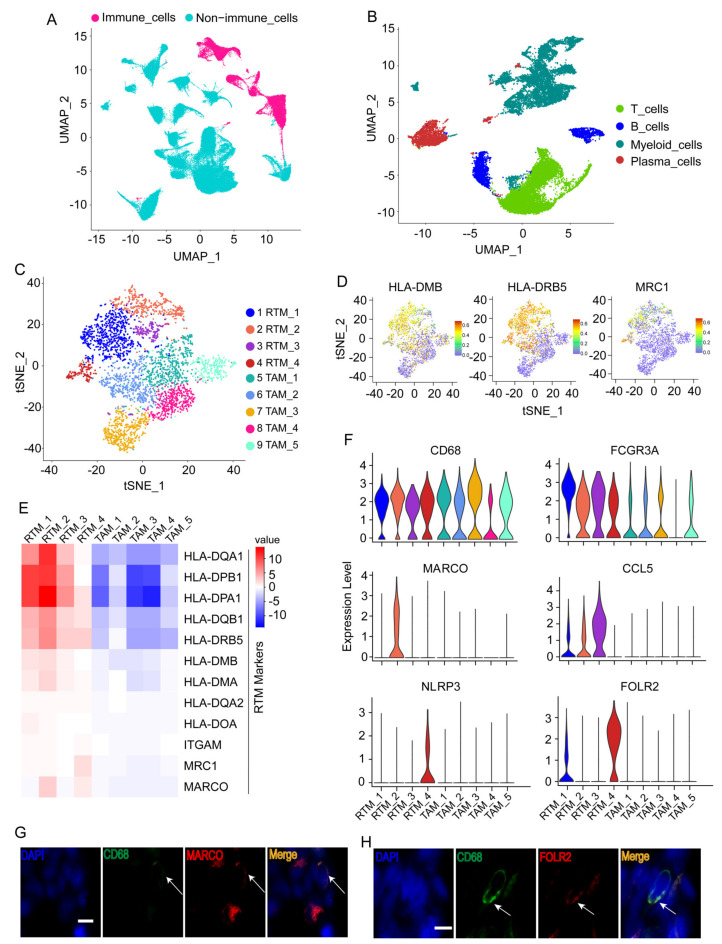
Identification of tissue-resident macrophages (RTMs). (**A**) UMAP plot of immune cells or non-immune cells. (**B**) UMAP plot of major immune cell types. (**C**) t-distributed stochastic neighbor embedding (t-SNE) plots displaying 10 macrophage clusters. (**D**) t-SNE plots showing the expression levels of representative genes. (**E**) Heatmap exhibiting the expression levels of representative genes across 10 macrophage clusters. RTM, tissue-resident macrophage; TAM, tumor-associated macrophages. (**F**) Violin plots showing the expression of RTM cluster-specific genes across five RTM clusters. RTM_1 cluster had no specific marker. (**G**,**H**) Immunofluorescence staining of CD68, MARCO (**G**), and FOLR2 (**H**) in BC tissues. Scale bar, 20 μm.

We utilized the Seurat package to perform fine clustering of the original single cells based on raw data from a previous BC study [10]. These cells were divided into immune cells (CD45^+^) and non-immune cells (CD45^-^) and were visualized by performing uniform manifold approximation and projection (UMAP) (Figure 1A). To further identify the cell subclusters of these immune cells, the immune cells were reclustered separately (Figure 1B), and cluster-specific genes were used to annotate cell types with classic markers documented in previous studies (Table S1).

Myeloid cells and their markers were exhibited by t-SNE plot (Figure S1A−D). In addition, myeloid cells from healthy breast tissues are demonstrated in the UMAP plot in Figure S2A,B. The myeloid cells in normal tissues are very different from those in BC tissues (Figure S2C). The proportions of major cell types, including myeloid cells, in healthy breast tissues and BC tissues were also exhibited (Figure S2D,E). Furthermore, all myeloid cells from BC tissues expressing macrophages markers (Figure 1C) were re-clustered separately to further identify the RTMs. The significant marker genes of each cluster are shown in Table S2. Five macrophage clusters (RTM_1-RTM_4) were consistent with the features of resident macrophages previously reported [3], including a high expression of MHCII (e.g., HLA-DMB, HLA-DRB5, HLA-DQA1, HLA-DOB, HLA-DPB1, HLA-DPA1, etc.), CD11b/ITGAM, and MRC1 (Figure 1D,E, and Figure S1B), and were denoted as RTMs, whereas the remaining clusters were denoted as tumor-associated macrophages (TAM_1-TAM_5). We revealed the expression profiles of RTM cluster-specific genes across five RTM clusters (Figure 1F). A high expression of FCGR3A was observed in all RTM clusters (Figure 1F). A previous study showed FCGR3 on myeloid cells was activated by agonistic CD40 antibodies, leading to the maturation of dendritic cells and activation of CD8+ T cells [20]. We also observed that the RTM_2 cluster uniquely expressed MARCO (Figure 1F). Previous studies demonstrated that MARCO was specifically expressed by macrophages [21] and mediated the clearance of tumor cells [22]. The RTM_3 cluster exhibited a high expression of CCL5 (Figure 1F). In a previous study, CCL5 was found to interact with CXCL9 expressed by macrophages, leading to an increase in T cell infiltration and inhibition of tumor progression [23]. In addition, the RTM_4 cluster revealed a unique NLRP3 expression and a high FOLR2 expression (Figure 1F). A recent study demonstrated that FOLR2+ macrophages positively correlated with CD8+ T cell infiltration [4], suggesting that the RTM_4 cluster exerted an anti-tumor activity in BC. Next, immunofluorescence staining was performed to prove the presence of these RTM clusters. As shown in Figure 1G,H, representative RTM-specific markers MARCO and FOLR2 were expressed in CD68+ macrophages (Figure 1G,H). Collectively, these results suggest RTMs play an important anti-tumor role in BC.

### 3.2. The Enrichment of Significant Pathways in RTMs

**Figure 2:**
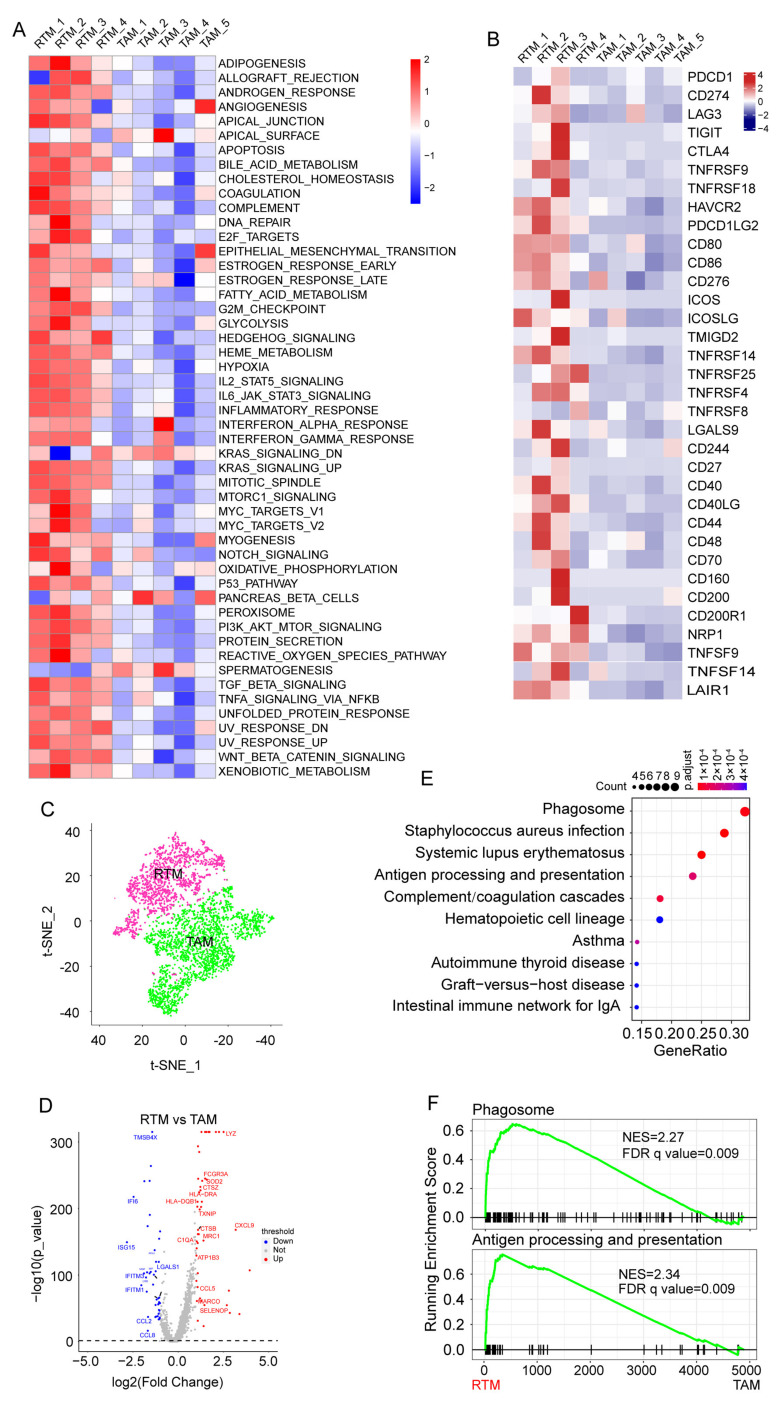
Enrichment of significant pathways in RTMs. (**A**) Differences in 50 hallmark pathway activities scored with GSVA software. The pathways highlighted were shown with red font. (**B**) Heatmap showing the expression of immune checkpoint molecules across 10 macrophage clusters. (**C**) The t-SNE plot of macrophages divided into RTM group and tumor-associated macrophages (TAM) group. (**D**) Volcano plot exhibiting key differentially expressed genes (DEGs) between RTMs and TAMs. (**E**) KEGG analysis of upregulated DEGs. (**F**) GSEA showing significant enrichment of phagosome pathway and antigen processing and presentation pathway in RTMs.

The gene set variation analysis (GSVA) for macrophages revealed that signal pathways were enriched in macrophage clusters, highlighting that all RTM clusters, i.e., RTM_1, RTM_2, RTM_3, and RTM_4, had similar features (Figure 2A). Remarkably, the inflammatory response and TNFα signaling, two important hallmarks of M1 macrophages as previously described [24,25], were enriched in each RTM cluster (Figure 2A). Meanwhile, we observed that the interferon response, Notch pathway, and TGFB signaling, which are three important hallmarks of M2-like macrophages as previously described [24,25,26,27], were enriched in each RTM cluster (Figure 2A). In agreement with these observations, we found all RTM clusters expressed M1 and M2 marker genes (Figure S1F). These results indicate that all RTM clusters express a unique mixed M1-M2 phenotype. These RTM clusters may be transitional cell types in the evolution from the M1 to M2 phenotype. Moreover, we found that each RTM cluster highly expressed immune checkpoint genes (Figure 2B). This result suggests that RTMs might be novel immunotherapeutic targets for advanced or recurrent BC.

Next, we categorized the macrophages into the TAM group and RTM group to investigate the function of RTMs in BC based on their analogous characteristics (Figure 2C). RTMs were highly distinct, with 4000 genes at a false discovery rate (FDR) of <1% differentially expressed compared with TAMs (Figure 2D and Table S3). The Kyoto Encyclopedia of Genes and Genomes (KEGG) and Gene Set Enrichment Analyses (GSEA) of differentially expressed genes (DEGs) showed that the phagosome pathway and antigen processing and presentation pathway are enriched in RTMs (Figure 2E,F). Collectively, these results indicate that RTMs exert an anti-tumor activity in BC. 

### 3.3. Immune Characteristics of Each RTM Cluster

**Figure 3:**
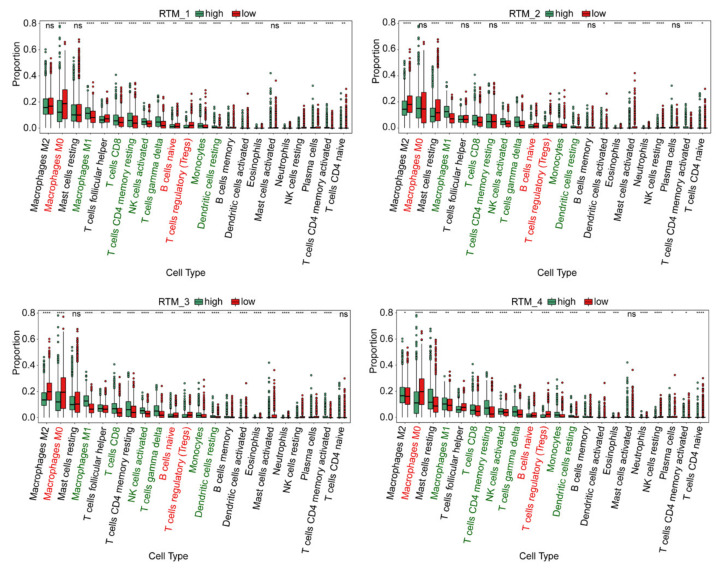
The immune characteristics of different subtypes of each RTM cluster. The proportions of immune cell clusters in different subgroups of each RTM cluster. The scattered dots represent the immune score of the two subgroups of each RTM cluster. The thick lines represent the median value. The bottom and top of the boxes are the 25 th and 75 th percentiles (interquartile range), respectively. M1 macrophages, CD8T cells, resting memory CD8T cells, activated NK cells, gamma delta T cells, monocytes and resting dendritic cells (green font) were more abundant in each RTM cluster-high subgroup, while M0 macrophages, naïve B cells and T regulatory cells (Tregs) (red font) were more abundant in each RTM cluster-low subgroup. Significant statistical differences between the two subgroups were assessed using the Wilcoxon test (ns: not significant, * *p* < 0.05, ** *p* < 0.01, *** *p* < 0.001, **** *p* < 0.0001).

To investigate the role of each RTM cluster in the tumor microenvironment of BC, we analyzed the correlations between two subtypes of each RTM cluster and 22 human immune cell subsets of every BC sample using the CIBERSORT algorithm. We found that M1 macrophages, CD8T cells, resting memory CD8T cells, activated NK cells, gamma delta T cells, monocytes and resting dendritic cells were more abundant in each RTM cluster-high subgroup, while M0 macrophages, naïve B cells and T regulatory cells (Tregs) were more abundant in each RTM cluster-low subgroup (Figure 3). We then applied certain gene signatures to define the immune function between different subgroups of each RTM cluster. As a result, there were more CD8T cells, activated NK cells and dendritic cells in the RTM cluster-high subgroup, while there were more immunosuppressive cells, such as Tregs, in the RTM cluster-low subgroup. In addition, we explored the relationship between TAM clusters and immune infiltration. We found that the effect of the TAM clusters on immune infiltration is different from that of RTM. Each TAM cluster-high subgroup was associated with the high infiltration of immunosuppressive cells, such as Tregs, while each TAM cluster-low subgroup was correlated with M2 macrophages and resting memory CD8T cells (Figure S3). Collectively, these results suggest RTM clusters may contribute to immunotherapy.

### 3.4. The Prognostic Analysis of Different RTM Clusters

**Figure 4:**
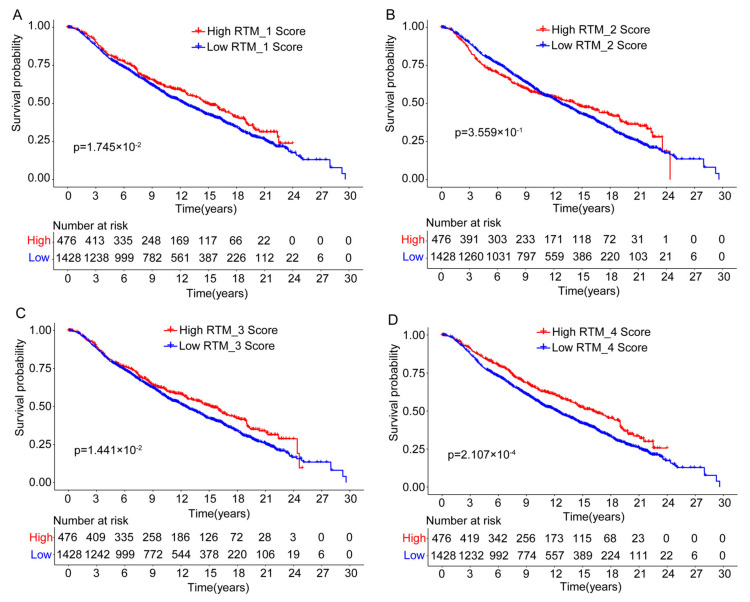
The prognostic association between RTM clusters and overall survival. (**A**–**D**) Kaplan-Meier survival analysis showed that all RTM clusters, excluding RTM_2 (**B**), were significantly correlated with improved overall survival in breast cancer patients. Split patients by upper quartile.

As our data suggest that RTM clusters contribute to the infiltration of immune cells, we predicted that RTM clusters would provide important prognostic information. Using the available gene expression data from the “METABRIC” consortium, we further evaluate the prognostic relevance of the RTM clusters identified in a setting free from external immunotherapeutic pressures. As shown in Figure 4A−E, we found that all RTM clusters, excluding the RTM_2 cluster, were significantly correlated with improved overall survival in BC patients. Prognosis in breast cancer has been associated with a higher tumor-infiltrating lymphocytes (TIL) level [28], and these results were consistent with the similar immunomodulatory functional roles of distinct RTM clusters. Furthermore, we explored the relationship between TAM clusters and prognosis using the METABRIC database. We found that the TAM_3 cluster tended to be associated with poor prognosis, while TAM_2, TAM_4, and TAM_5 were correlated with improved prognosis (Figure S4).

### 3.5. RTM Clusters Are Associated with Sensitivity to ICT 

**Figure 5:**
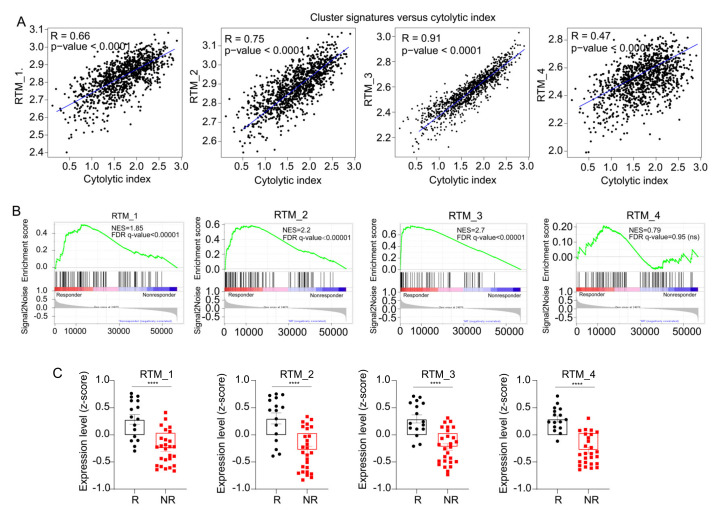
RTM clusters are associated with immune checkpoint therapy. (**A**) Correlation curves between RTM gene signatures and cytolytic index in TCGA cohort. (**B**) Analysis of bulk RNA-seq data of 43 breast cancer samples before ICT treatment using the GSEA. Results showed that gene signatures from each RTM cluster (see Table S4), excluding RTM_4, were significantly enriched in responders (n = 16) compared with nonresponders (n = 27). (**C**) Expression assessed by average z-score of each RTM cluster signature in responding (R) and nonresponding (NR) patients with BC. **** *p* < 0.0001, measured with unpaired *t* test.

A recent study on the positive relevance of RTMs to CD8+ T cells infiltration [4] suggested RTMs could enhance ICT response. As shown in Figure 5A, we observed a positive correlation between the T-cell cytolytic index [29] and gene signatures of each RTM cluster (gene signatures of each cluster are shown in Table S4). The BC patient response categories were defined by RECIST (response evaluation criteria in solid tumors) as following: complete response (CR) and partial response (PR) for responders (R), or stable disease (SD) and progressive disease (PD) for non-responders (NR). By performing GSEA, we observed that specific gene signatures from each RTM cluster, excluding the RTM_4 cluster, were significantly enriched in responder patients (n = 16) compared with nonresponder patients (n = 27) (Figure 5B). Representative genes for each category are shown in Table S7. We next compared the content in each RTM cluster in responders versus nonresponders. We confirmed that each cluster expression was significantly higher in responders than in nonresponders (Figure 5C). In conclusion, these results indicate that the identified RTM clusters are associated with sensitivity to ICT.

### 3.6. The Development of an ICT Outcome Signature

**Figure 6:**
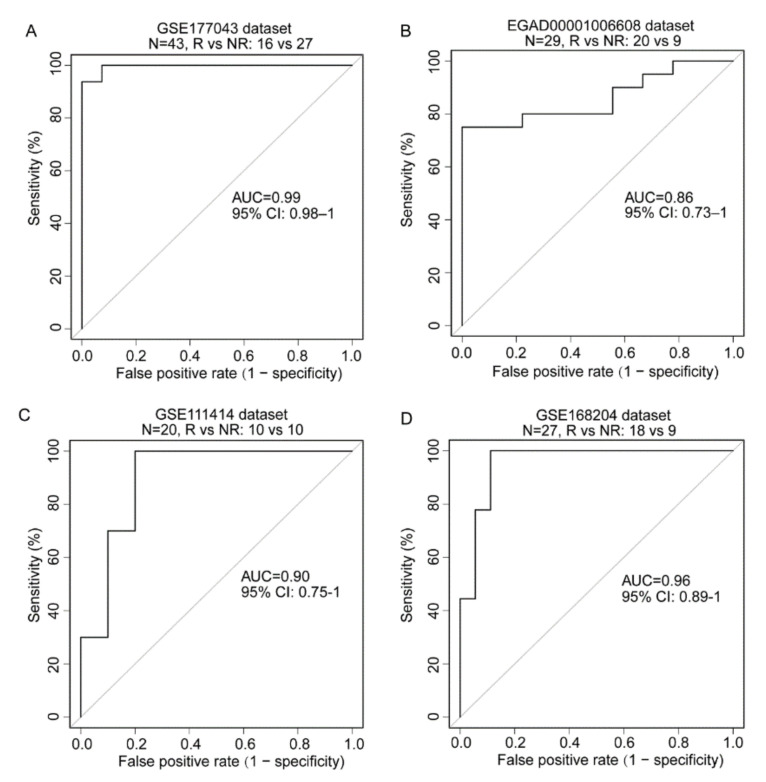
Prediction of ICT outcomes by RTM.Sig. (**A**) The RTM.Sig accurately predicted ICT outcomes of BC patients in initial discovery dataset (accession number: GSE177043). The RTM.Sig had an AUC value of 0.99 (95% CI: 0.98–1). (**B**) The predictive performance of RTM.Sig in the first validation dataset (accession number: EGAD00001006608) was as follow: AUC value of 0.86 (95% CI: 0.73–1). (**C**) The performance of RTM.Sig in predicting ICT outcomes in the second validation dataset (accession number: GSE111414) was shown. The RTM.Sig had an AUC value of 0.90 (95% CI: 0.75–1). (**D**) The predictive performance of RTM.Sig was displayed in the third validation dataset (accession number: GSE168204). The RTM.Sig had an AUC of 0.96 (95% CI: 0.89–1).

Because each RTM cluster expression and enrichment of gene signatures from each RTM cluster were significantly higher in responding patients than in nonresponding patients, we hypothesized that the expression of the feature genes of these clusters may predict ICT outcome. To verify this hypothesis, we developed an ICT responsiveness signature (Table S5) based on the scRNA-seq dataset and a bulk gene expression dataset—GSE177043—using the cancerclass R package [30]. This signature was denoted as an RTM signature (RTM.Sig) and had significantly high prognostic values for ICT outcomes. Specifically, for the initial discovery dataset—GSE177043 (N = 43, Responder [R] vs. Nonresponder [NR]: 16 vs. 27), the RTM.Sig had an (area under the curve) AUC of 0.99 (95% confidence interval [CI], 0.98–1), sensitivity of 100% (95% CI: 100–100%), and specificity of 92.59% (95% CI: 81.48–100%) (Figure 6A). For the validation dataset—EGAD00001006608 (N = 29, R vs. NR: 20 vs. 9)— the RTM.Sig also accurately predicted ICT outcomes of BC patients, with an AUC of 0.86 (95% CI: 0.73–1), sensitivity of 80% (95% CI: 60–95%), and specificity of 78% (95% CI: 44–100%) (Figure 6B). 

For further validation, we downloaded and analyzed GSE111414 and GSE168204 datasets. These datasets comprised the gene expression profile with clinical information on anti-PD-1 immunotherapy. For the GSE111414 dataset (N = 20, R vs. NR: 10 vs. 10), the pretreatment samples were selected for validation. RTM.Sig performed efficiently in distinguishing NR from R tumors with an AUC of 0.90 (95% CI, 0.75–1), sensitivity of 90% (95% CI: 70–100%), and specificity of 80% (95% CI: 50–100%) (Figure 6C). For the GSE168204 dataset (N = 27, R vs. NR: 18 vs 9), the pretreatment tumor samples were selected for validation. The RTM.Sig precisely predicted ICT outcomes with an AUC of 0.96 (95% CI, 0.89–1), sensitivity of 100% (95% CI: 100–100%), and specificity of 89% (95% CI: 72–100%) (Figure 6D). These results show that RTM.Sig can accurately predict ICT outcomes across all four independent datasets.

**Figure 7:**
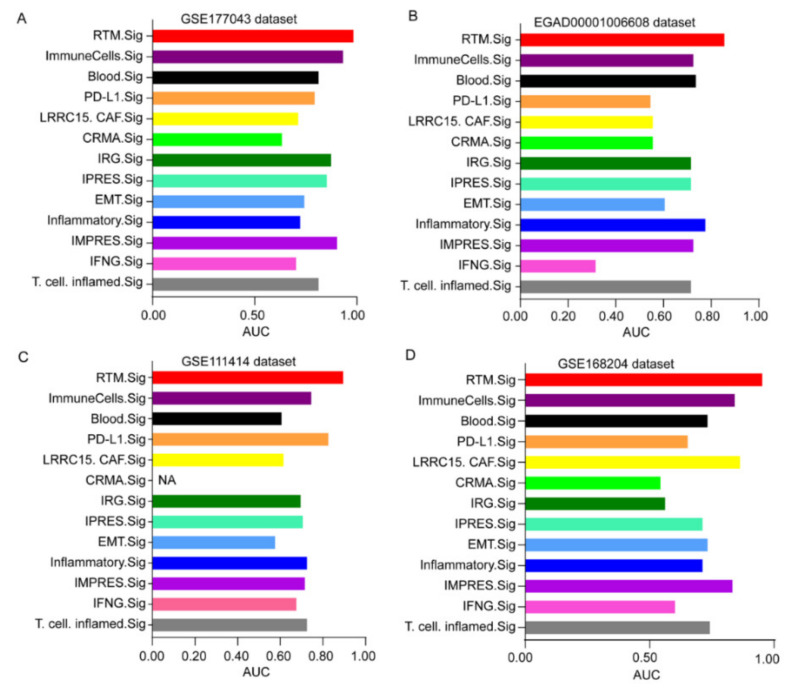
Comparing the predictive performance of RTM.Sig with previous gene signatures. Multiple bar plots showing the AUC values of 13 ICT response signatures in the GSE177043 dataset (**A**), in the EGAD00001006608 dataset (**B**), in the GSE111414 dataset (**C**) and in the GSE168204 dataset (**D**). NA (not available) means this gene signature was not found in corresponding dataset.

Next, we further compared the predictive performance of RTM.Sig with the other gene signatures reported previously (Table 1) [19,31,32,33,34,35,36,37,38,39,40]. The result showed that the performance of RTM.Sig in predicting response to ICT was consistently the best across all four datasets. As a reference, the well-established IMPRES was ranked 3rd in prediction accuracy in the GSE177043 dataset (Figure 7A and Figure S5), 5th in the EGAD00001006608 dataset (Figure 7B and Figure S6), 6th in the GSE111414 dataset (Figure 7C and Figure S7), and 4th in GSE168204 dataset (Figure 7D and Figure S8). This shows that RTM.Sig is the best biomarker for the prediction of ICT outcomes across four independent datasets.

## 4. Discussion

Breast cancer (BC) is a common malignant tumor with a high relapse rate. Immune checkpoint therapy (ICT) is a gold standard therapy for advanced-stage BC. Nevertheless, the majority of patients do not respond to ICT, resulting in treatment failure. The discovery of a gene expression signature in predicting ICT response is a valuable tool for managing patients under ICT. Increasing evidence indicates that infiltrating lymphocytes and myeloid cells in the tumor immune microenvironment affect ICT outcomes. As such, investigating the immune microenvironment of BC at the single-cell level will help in identifying novel therapeutic approaches for ICT. Herein, we found that RTMs precisely predict the ICT outcomes of BC patients compared with the previously reported signatures, thereby providing an important tool for clinical use.

We performed scRNA-seq analysis and identified five RTM clusters from BC samples. We observed that inflammatory response and TNFα signaling, two important hallmarks of M1 macrophages as described in previous studies [24,25], were enriched in each RTM cluster. Meanwhile, we observed that the IFN pathway, Wnt signaling, and Notch pathway, which are three hallmarks of M2 macrophages [24,25,26], were enriched in each RTM cluster. These results show that RTM clusters exhibit a mixed phenotype of M1-M2 macrophages, and hence might be a transitional cell type of M1 and M2 macrophages. A recent study revealed that RTMs promotes CD8+ T cell infiltration [4], agreeing with our findings that RTMs are directly proportional with T-cell cytotoxicity. 

We observed high FCGR3A expression in all RTM clusters. The previous studies demonstrated that FCGR3 on myeloid cells promoted CD40 antibody-mediated maturation of dendritic cells and activation of CD8+ T cells, and thereby drove the anti-tumor activity of agonistic CD40 antibodies [20]. We also observed a unique MARCO expression in the RTM_2 cluster. MARCO was previously reported as a restricted expression profile in lymph nodes, spleen, lung, peritoneum, and activated dendritic cells [41,42]. In addition, the RTM_3 cluster exhibited a high CCL5 expression. A previous study demonstrated that CCL5 promoted T cell infiltration and inhibited tumor progression [13]. The RTM_4 cluster revealed a unique NLRP3 expression and a high FOLR2 expression. FOLR2+ macrophages have been demonstrated to promote CD8+ T cell infiltration [4], which suggests that the RTM_4 cluster exerted an anti-tumor activity in BC. Collectively, our present results indicate that RTM clusters play an antitumor role in BC.

Given that each RTM cluster expression and enrichment of gene signatures from each RTM cluster were significantly higher in responding patients than in nonresponding patients, we developed a 25-gene expression signature—RTM.Sig—enriched with the genetic characteristics of RTM clusters to predict response to ICT. We demonstrated that the RTM.Sig can accurately predict the ICT outcomes of BC patients across two independent datasets compared with previously reported ICT response signatures. Our characterization of RTM clusters provides effective biomarkers in predicting immunotherapy response and the novel targets that improve the efficacy of ICT.

## 5. Conclusions

In this study, we developed a 25-gene signature based on tissue-resident macrophages—RTM.Sig—to predict response to immunotherapy in breast cancer patients. RTM.Sig can more accurately predict ICT outcomes of breast cancer patients relative to previous outstanding signatures. These findings advance our understanding of RTMs, and may be used to improve clinical diagnosis and treatment strategies for BC.

## Figures and Tables

**Table 1:** The list of biomarkers for predicting response to immune checkpoint therapy compared in this study.

Signature ID	Description
RTM.Sig	The tissue-resident macrophages (RTM) signature identified in this study
ImmuneCells.Sig	A 108-gene expression signature predicted response to immune checkpoint therapy in melanoma [19]
IPRES.Sig	A 16-gene expression signature predicted response to anti-PD-1 antibody therapy in melanoma [31,32]
EMT.Sig	A gene expression signature consisted of 12 epithelial-mesenchymal transition (EMT)-related genes predicted immunotherapy response in lung cancer [32]
CRMA.Sig	A 5-gene expression signature, including MAGEA2, MAGEA2B, MAGEA3, MAGEA6, and MAGEA12, predicted immunotherapy response in melanoma [33]
Inflammatory.Sig	A gene expression signature based on 27 inflammation related genes can provide prediction of immune checkpoint blockade response in lung cancer [32]
IFNG.Sig	A 6-gene biomarker of interferon gamma (IFNγ) response, including IFNG, STAT1, IDO1, CXCL10, CXCL9, and HLA-DRA, can predict immunotherapy response [34]
IRG.Sig	A prognostic signature containing 11 immune-related genes (IRGs) for predicting ICT outcomes of CC (cervical cancer) patients [35]
Blood.Sig	A 15-gene expression signature derived from blood sample that provided prediction to anti-CTLA4 immunotherapy response in melanoma [36]
PD-L1.Sig	A gene signature including PD-L1, PD-L2,and PD-1 [37]
IMPRES.Sig	Immuno-predictive score (IMPRES), a predictor of Immune checkpoint blockade (ICB) response, can predict response to ICT outcomes of melanoma patients [38]
LRRC15.CAF.Sig	A specific type of carcinoma-associated fibroblasts (CAF) signature, i.e., LRRC15 + CAF, can predict anti-PD-L1 therapy resistance [39]
T.cell.inflamed.Sig	An 18 gene “T-cell–inflamed gene expression signature” that predicted clinical outcomes of ICT in various cancer types [33,40]

## Data Availability

All datasets used in this study are available in a public, open access repository. The scRNA-seq dataset is available on the GEO database under accession number GSE161529. The METABRIC dataset was obtained from cBioPortal website (http://www.cbioportal.org, (accessed on 25 August 2022)). Four bulk RNA-seq datasets of patients treated with immunotherapy were obtained from GEO (numbers: GSE177043, GSE111414 and GSE168204) and European Genome-phenome Archive (numbers: EGAD00001006608). All other relevant data are available in this article and Supplementary Information.

## Supplementary Materials

The following supporting information can be downloaded at: https://www.mdpi.com/article/10.3390/cancers14225506/s1, Figure S1: The features of tissue-resident macrophages; Figure S2: Identification of myeloid cells from healthy breast tissue; Figure S3: The immune characteristics of different subtypes of each TAM cluster; Figure S4: The prognostic association between TAM clusters and overall survival; Figure S5: The AUC values of other ICT response signatures in GSE177043 dataset; Figure S6: The AUC values of other ICT response signatures in EGAD00001006608 dataset; Figure S7: The AUC values of other ICT response signatures in GSE111414 dataset; Figure S8: The AUC values of other ICT response signatures in GSE168204 dataset; Table S1: Canonical marker genes used for cell type identification and relevant literatures; Table S2: Significant marker genes of macrophage subsets; Table S3: The list of differentially expressed genes (DEGs) between RTM and TAM; Table S4: Gene signatures from each RTM subset; Table S5: The identified RTM.Sig in this study; Table S6: The website summary of all R packages used in this study. Table S7: Representative genes for each category.
