# Supplementary material for: Integrated Analysis of Single-Cell and Bulk RNA-Sequencing Reveals a Tissue-Resident Macrophage-Related Signature for Predicting Immunotherapy Response in Breast Cancer Patients
Source: Cancers (Basel). 2022 Nov 9;14(22):5506. doi: 10.3390/cancers14225506 (PMC9688720; doi:10.3390/cancers14225506)
Supplement: Supplementary file 1 [file cancers-14-05506-s001.zip › Supplementary_materials.pdf]

Supplementary Materials for

Integrated analysis of single-cell and bulk RNA-sequencing reveals a tissue-resident macrophage-related signature for predicting immunotherapy response in breast cancer patients

Zi-An Xia, You Zhou, Jun Li, Jiang He*

*Corresponding author. Email: hj2008s@csu.edu.cn

This file includes:

Supplementary Text
Figures S1 to S8
Descriptions of Table S1 to S7

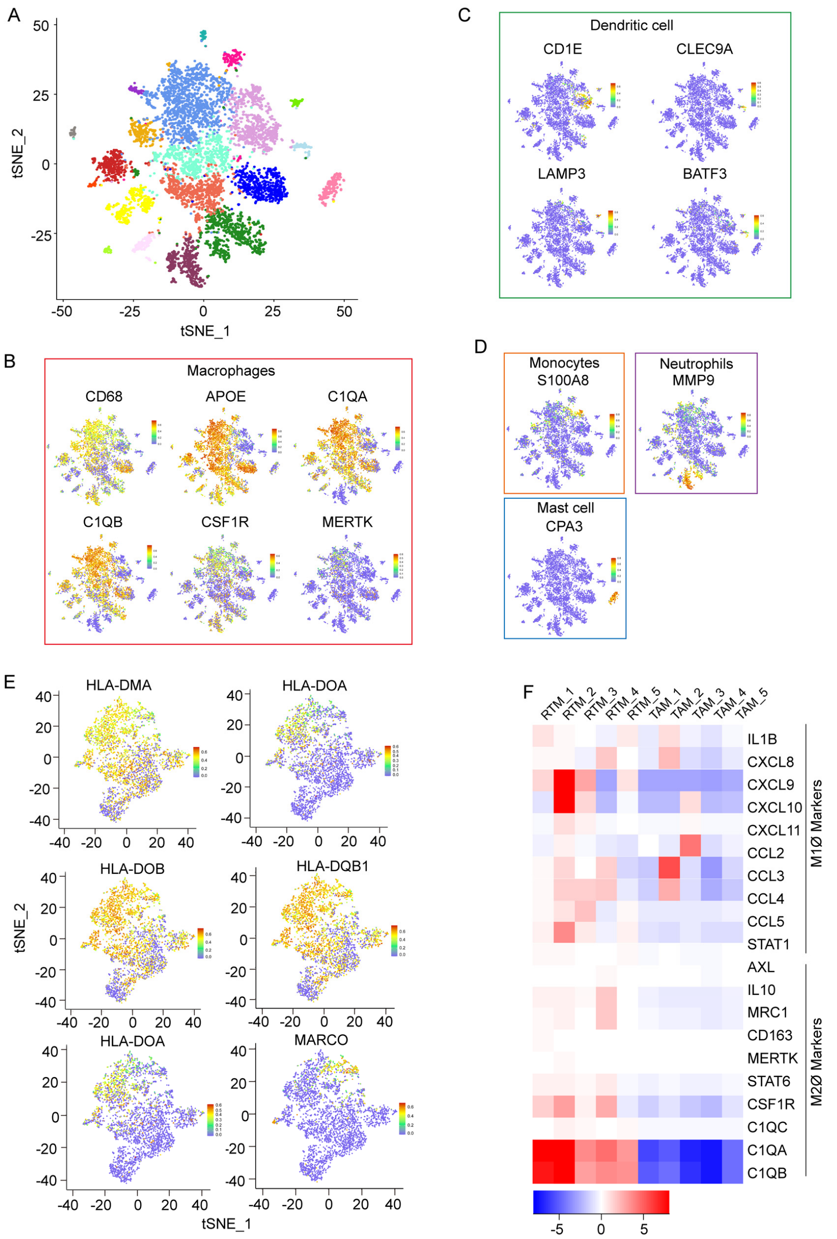

Figure S1. The features of tissue-resident macrophages. (A) tSNE plot showing myeloid cells from breast cancer. (B) t-SNE plots showing the expression of macrophage marker genes. (C) t-SNE plots exhibiting the expression of marker genes from dendritic cells. (D) t-SNE plots showing the expression of marker genes from monocytes, neutrophils, and mast cells respectively. (**E**) t-SNE plots for marker gene of tissue-resident macrophages (RTM). (**F**) The expression of marker genes of M1Ø or M2Ø was exhibited by heatmap.

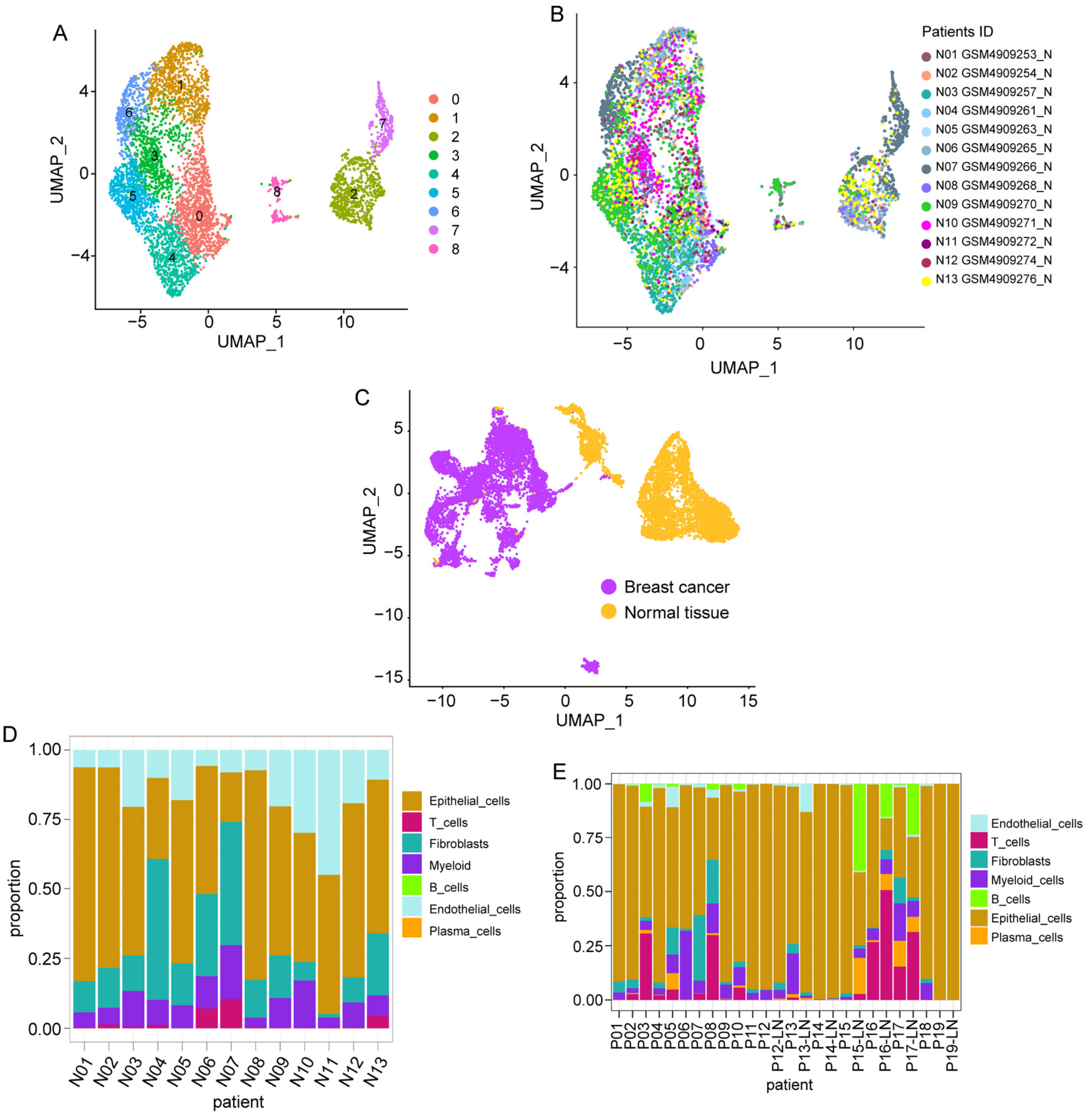

Figure S2. Identification of myeloid cells from healthy breast tissue. (A) UMAP plot showing myeloid cells from healthy breast tissues. (B) UMAP plot of myeloid cells from healthy breast tissues, colored by patients. (C) UMAP plot exhibiting myeloid cells from healthy breast tissues and breast cancer tissues. (D) Major cell-types composition of healthy breast tissues. (E) Major cell-types composition of breast cancer tissues.

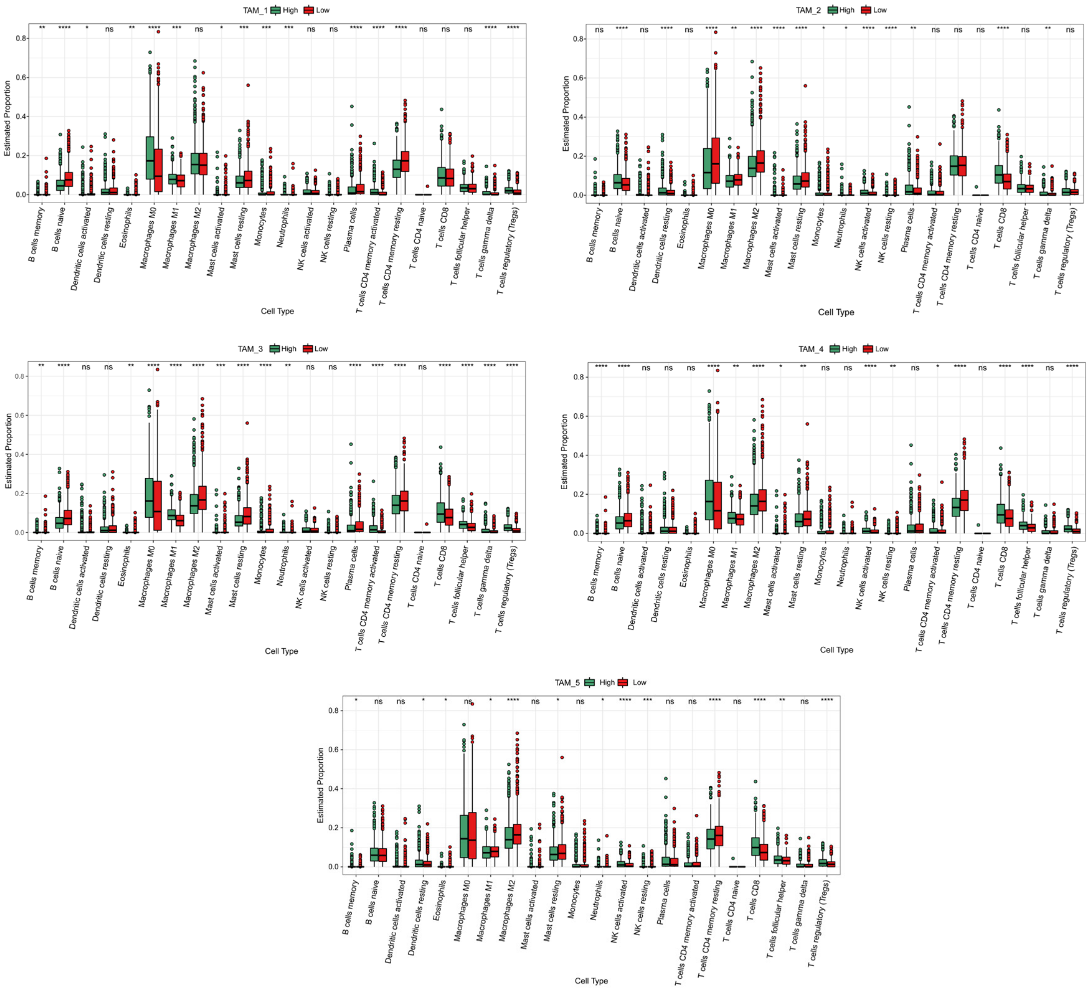

Figure S3. The immune characteristics of different subtypes of each TAM cluster. The proportions of immune cell clusters in different subgroups of each TAM cluster were exhibited. The scattered dots represent the immune score of the two subgroups of each TAM cluster. The thick lines represent the median value. The bottom and top of the boxes are the 25th and 75th percentiles (interquartile range), respectively. CD8 T cells, activated NK cells, gamma delta T cells, and T regulatory cells (Tregs) were more abundant in the each TAM cluster-high subgroup, while M2 macrophages, naïve B cells and resting memory CD4 T cells were more abundant in the each TAM cluster-low subgroup. Significant statistical differences between the two subgroups were assessed using the Wilcoxon test (ns: not significant, *p < 0.05, ** p< 0.01, *** p < 0.001, **** p < 0.0001).

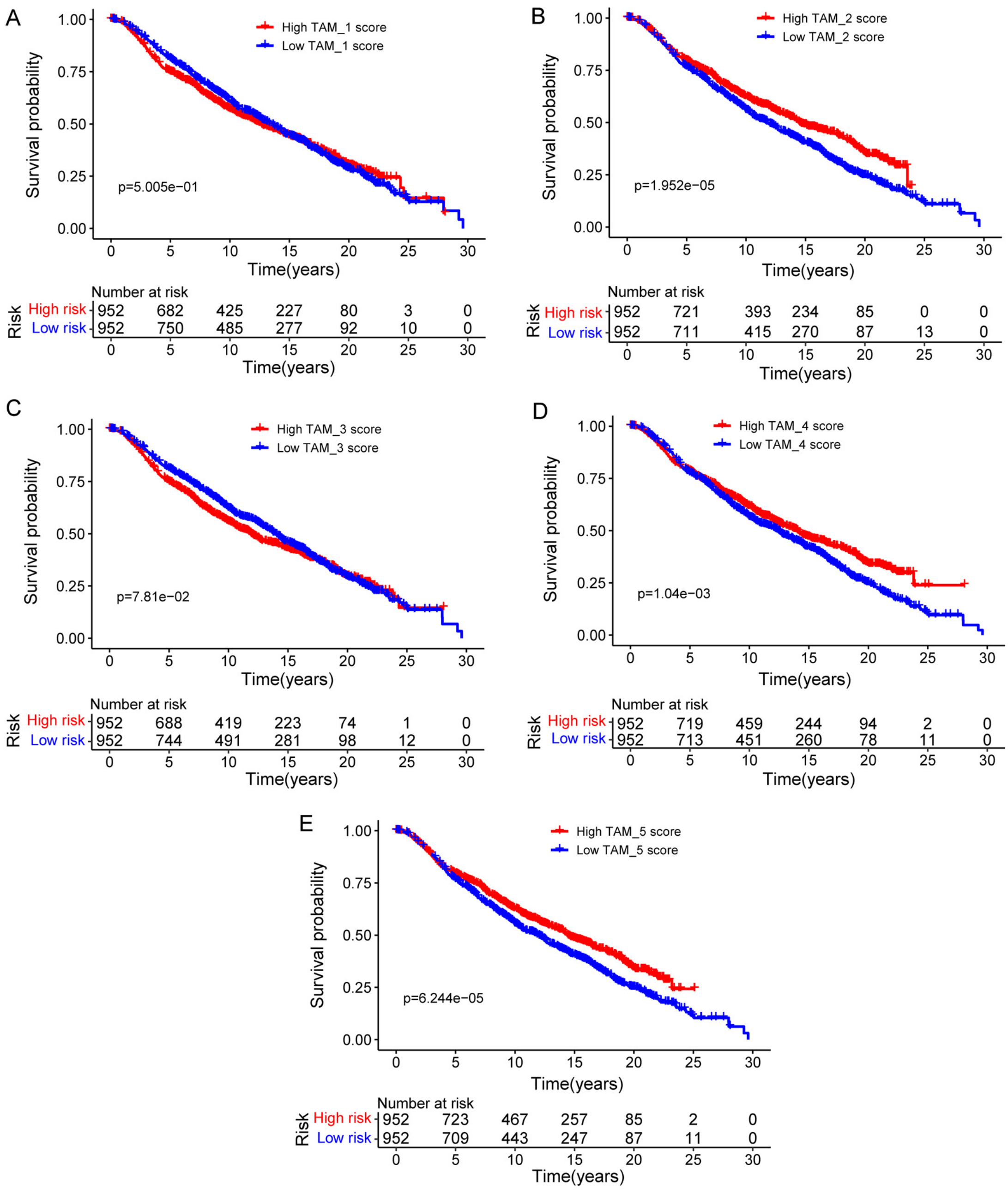

Figure S4. The prognostic association between TAM clusters and overall survival. (**A-E**) Kaplan-Meier survival analysis showed TAM_1 (**A**) and TAM_3 (**C**) subsets were not correlated with improved overall survival in breast cancer patients, while TAM_2, TAM_4, and TAM_5 subsets were significantly associated with prolong overall survival.

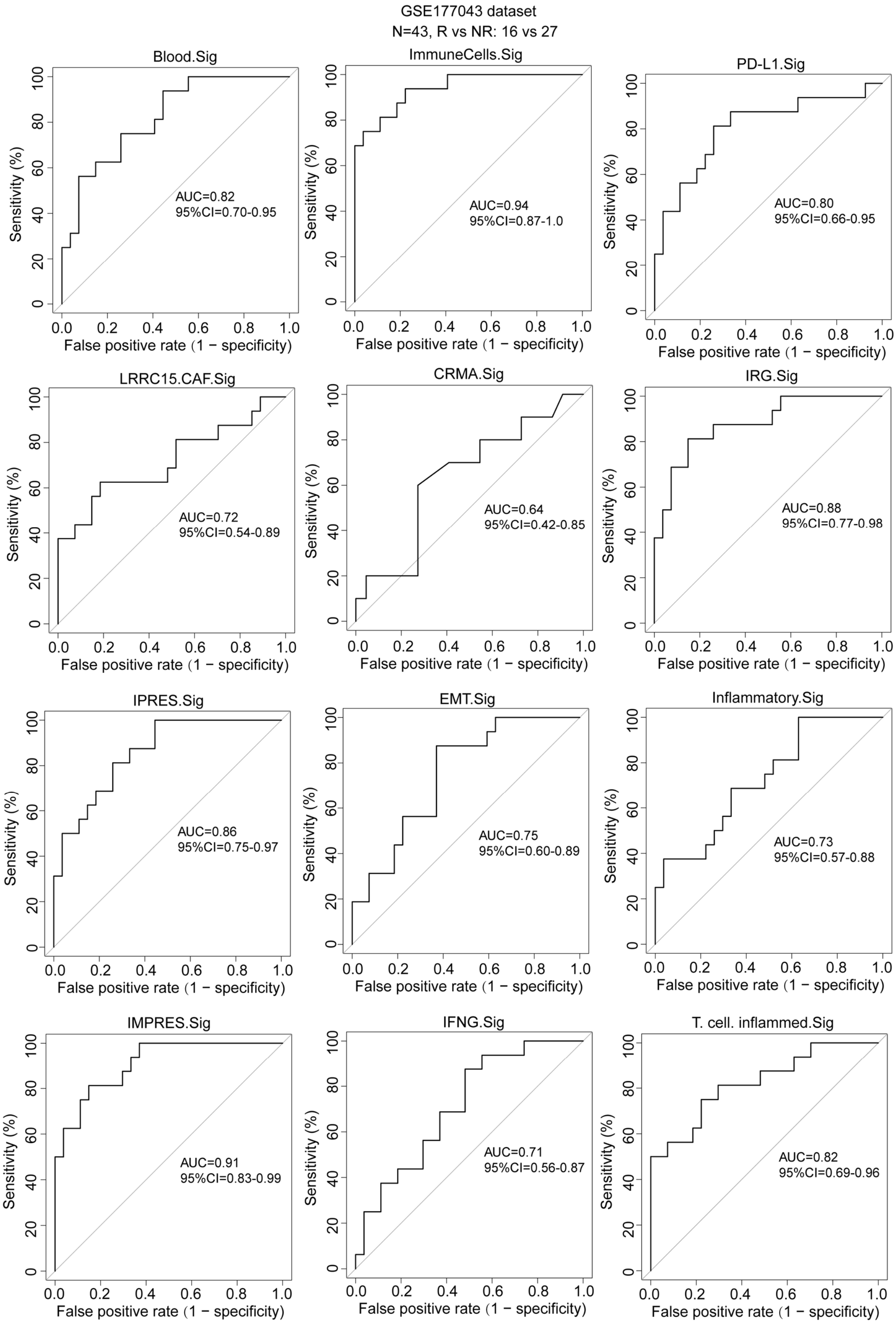

Figure S5. The AUC values of other ICT response signatures in GSE177043 dataset.

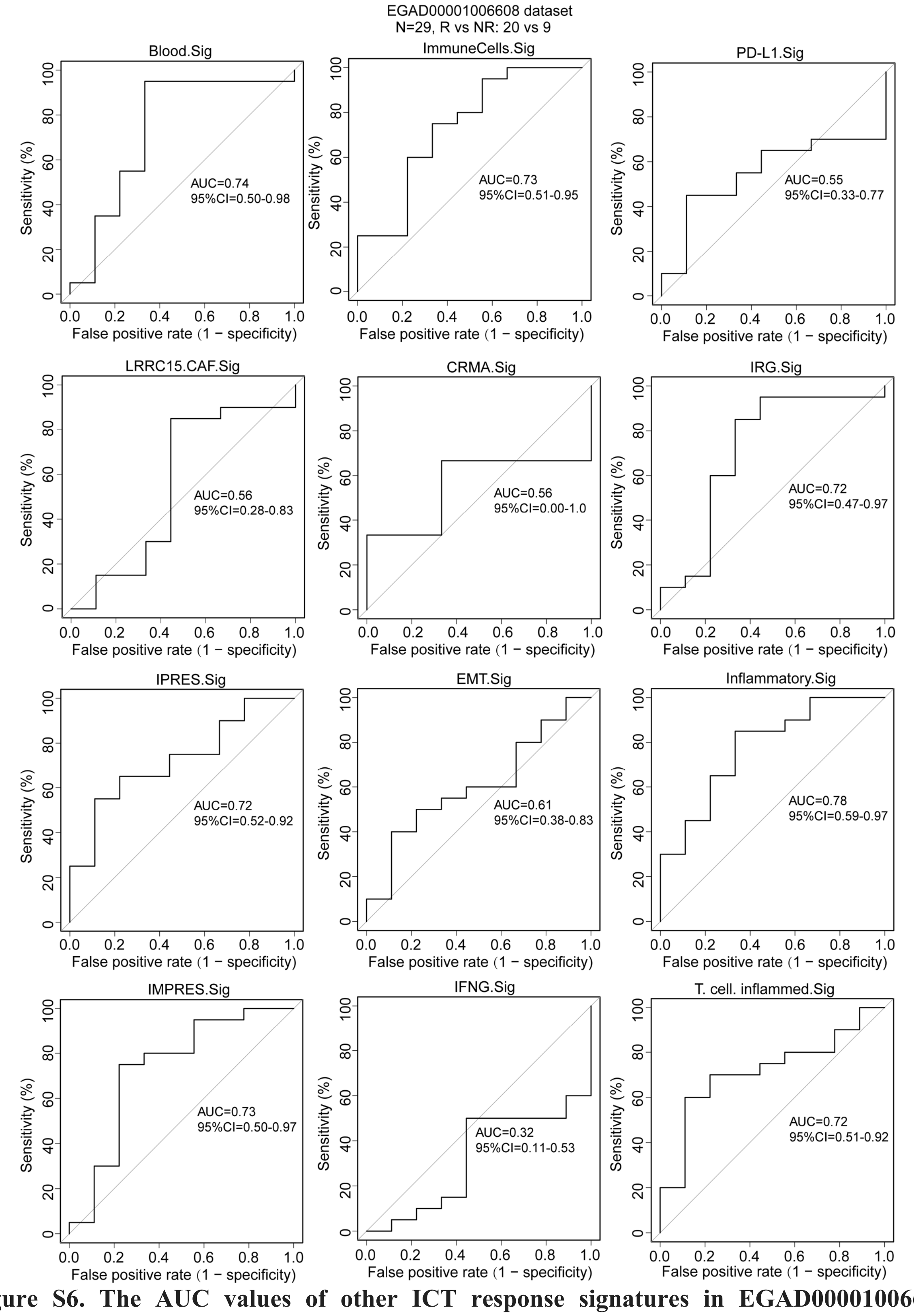

Figure S6. The AUC values of other ICT response signatures in EGAD00001006608 dataset.

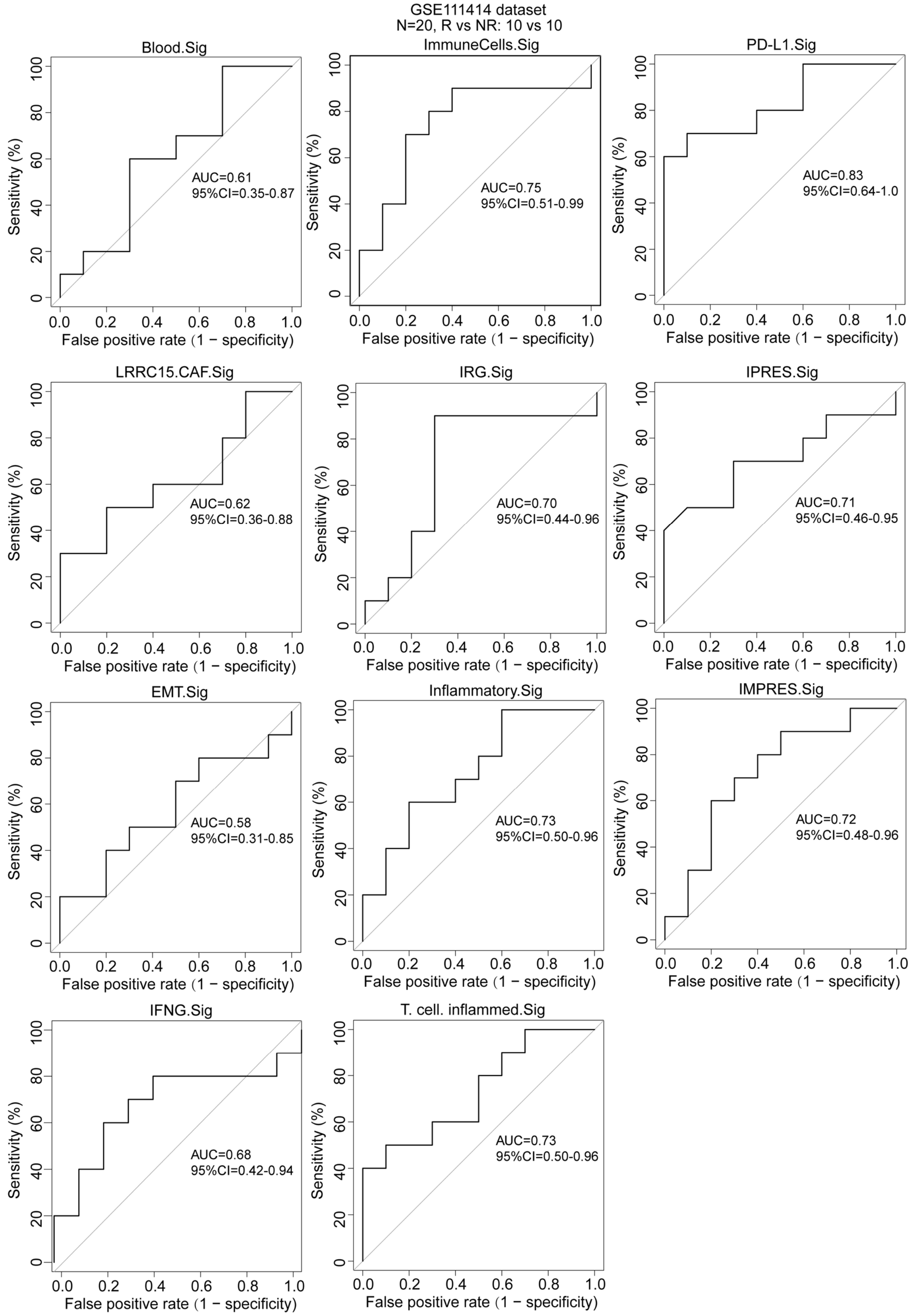

Figure S7. The AUC values of other ICT response signatures in GSE111414 dataset.

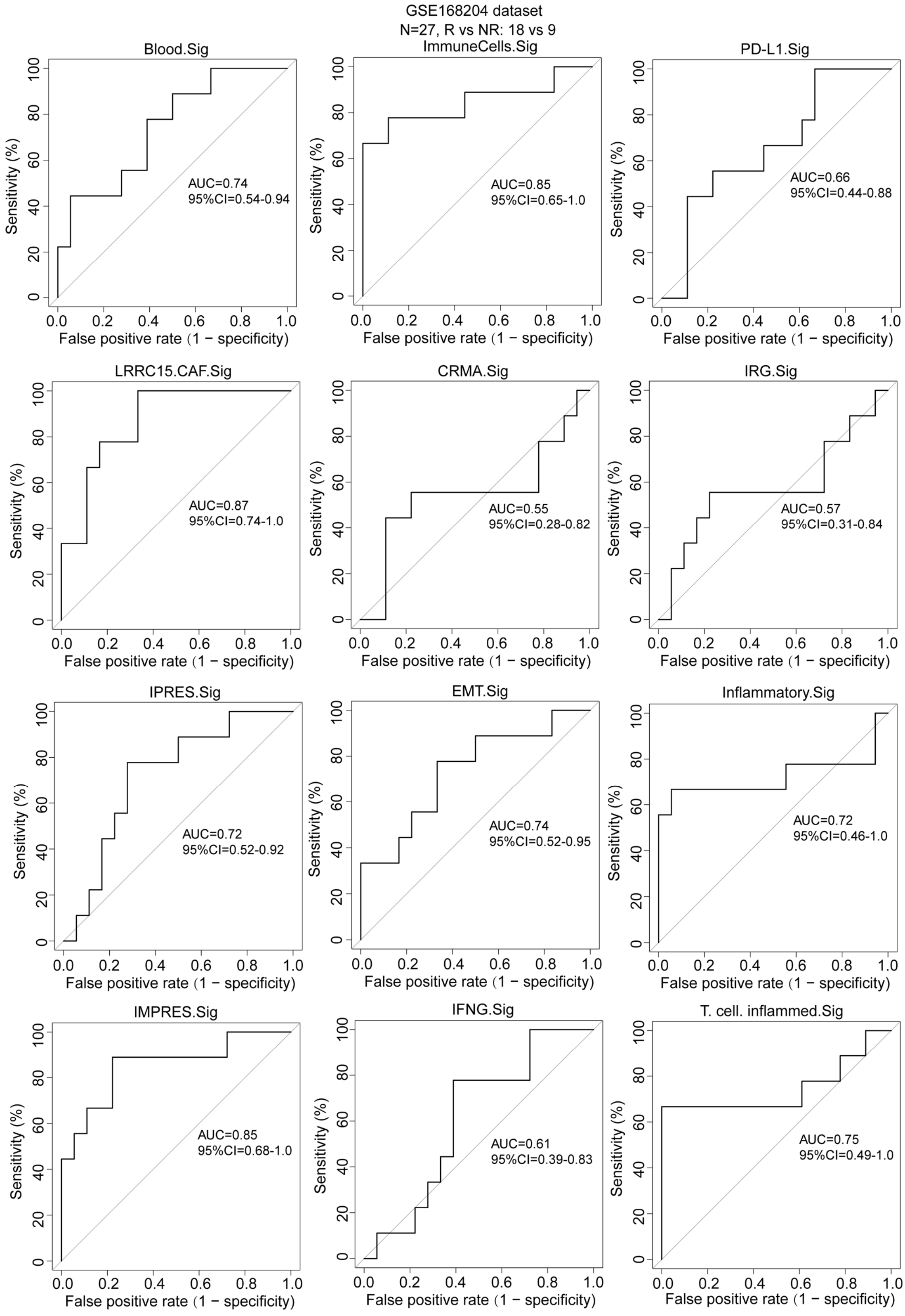

Figure S8. The AUC values of other ICT response signatures in GSE168204 dataset.

Table S1 Canonical marker genes used for cell type identification and relevant literatures, and molecular characterize/ stratify of BC samples.

Table S2 Significant marker genes of macrophage subsets.

Table S3 The list of differentially expressed genes (DEGs) between RTM and TAM.

Table S4 Gene signatures from each RTM subset.

Table S5 The identified RTM.Sig in this study

Table S6 The website summary of all R packages used in this study.

Table S7 Representative genes for each category
